# Piezo1 in digestive homeostasis and disease: Barrier function, immune signalling, cellular injury and metabolism

**DOI:** 10.1002/ctm2.70832

**Published:** 2026-09-14

**Authors:** Yuan Xia, Chang Liang, Zhuoqing Zhuang, Pingrun Chen, Zhe Xiong, Junzhu Lu, Yan Zhang

**Affiliations:** ^1^ Department of Gastroenterology and Hepatology West China Hospital, Sichuan University Sichuan Province China

**Keywords:** barrier homeostasis, digestive diseases, inflammation, mechanotransduction, metabolism, piezo1

## Abstract

**Background:**

Piezo1 is a mechanosensitive cation channel that converts stretch, compression, and shear stress into calcium‐dependent signals. This narrative review evaluates how Piezo1 contributes to digestive physiology and disease and why divergent outcomes arise across experimental contexts.

**Main body:**

We searched PubMed/MEDLINE, Embase, Web of Science, and Scopus from database inception to 15 July 2026, prioritising primary studies that used Piezo1‐specific genetic or pharmacological interventions. Piezo1‐specific evidence supports roles in epithelial junctional adaptation, mucus production, stem‐cell renewal, immune activation, injury, fibrosis, and gastrointestinal endocrine signalling. A contextual framework based on cell identity and state, mechanical stimulus, and tissue or disease phase helps explain these divergent effects. Transient physiological activation may support homeostasis and repair, whereas persistent or excessive activation can promote Ca^2+^ overload, inflammasome signalling, mitochondrial dysfunction, and matrix remodelling. The strongest digestive‐system evidence for mechanometabolic coupling concerns macrophage glycolysis, enterocyte nutrient handling, and selected endocrine and lipid‐regulatory pathways. Evidence for Piezo1‐dependent lactate production remains model‐specific, and direct digestive evidence for lactylation is absent. One mechanistic mouse study supports hepatic endothelial regulation of bile‐acid synthesis, whereas intestinal Piezo1 control of bile‐acid homeostasis remains unestablished.

**Conclusion:**

Therapeutic translation is limited by non‐selective tool compounds, broad tissue expression and the need for targeted delivery and phase‐appropriate modulation. Piezo1 is therefore best viewed as a context‐dependent mechanotransducer rather than a uniformly pathogenic target.

## Highlights

- Piezo1 responses are shaped by mechanical input, cell identity and state, pathological stress and disease phase—not channel activation alone.
- Transient, phase‐appropriate signalling supports homeostasis and repair, whereas sustained activation promotes inflammation, injury and remodelling.
- Piezo1 links barrier, immune, metabolic and remodelling responses as a context‐dependent mechanotransducer, not a uniformly protective, pathogenic, or universally actionable target.

## Graphical abstract

Piezo1 functions as a context‐dependent mechanotransduction node linking mechanical cues to inflammation, tissue remodelling and metabolic regulation in digestive homeostasis and disease. In epithelial, immune, stromal and endocrine cells, Piezo1‐mediated Ca^2+^ signalling contributes to barrier and mucus homeostasis, epithelial renewal and repair, inflammatory amplification, cellular stress and injury and fibrotic or tumour‐associated remodelling. Piezo1 also links gastrointestinal mechanical stimulation to nutrient handling, endocrine mechanosensing, macrophage glycolysis and selected lipid‐regulatory pathways. These effects vary according to mechanical input characteristics, cell identity and state, inflammatory or metabolic stress, and disease phase, allowing Piezo1 to support homeostasis and post‐injury repair in some contexts while promoting inflammation, tissue injury, fibrosis and tumour microenvironmental change in others.

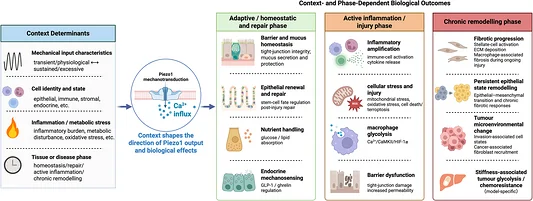

## 1 INTRODUCTION

Piezo1 is a mechanosensitive cation channel that has attracted growing interest in mechanobiology. It translates diverse mechanical cues into Ca^2+^‐centred intracellular signals.^1^ As the digestive system is continuously subjected to mechanical forces generated by luminal contents and gastrointestinal motility, mechanosensing is essential for maintaining structural and functional homeostasis.^2^, ^3^ Recent studies indicate that Piezo1 activation and gating are influenced not only by external mechanical forces but also by the local membrane lipid milieu, cholesterol and the cytoskeleton. These findings suggest that Piezo1 function is regulated at multiple levels and is not determined solely by immediate mechanical changes.^4^

In the gastrointestinal system, mechanical sensing and signal transduction contribute to digestion, absorption, secretion and epithelial barrier maintenance. Dysregulation of these pathways may contribute to disease development and progression. Evidence suggests that activated Piezo1 contributes to epithelial barrier homeostasis, secretory function, gastrointestinal motility, and inflammatory regulation through Ca^2+^‐dependent signalling pathways. Accordingly, Piezo1 is increasingly understood not merely as a mechanosensitive responder but also as a regulator of mucosal homeostasis and its disruption.^2^, ^3^ Piezo1 dysregulation has also been associated with mucus‐layer alterations and remodelling of the gut microbiota.^5^, ^6^ In macrophages, Piezo1 has been linked to inflammatory amplification and glycolytic activation.^7^ Broader studies of intestinal mechanobiology and mucosal immunity provide additional physiological context for these findings.^8^, ^9^, ^10^ The mucus layer and goblet cells are major components of the intestinal barrier and key interfaces linking mechanical stimuli, epithelial homeostasis and the spatial organization of the gut microbiota. Structural and functional abnormalities in these components may precede or accompany inflammation and further contribute to mucosal immune alterations and local microenvironmental remodelling.^6^, ^8^ In digestive diseases, Piezo1 may thus provide an important mechanistic entry point for linking aberrant mechanical stimuli with mucosal pathological responses. This mechanotransduction‐based perspective may help reinterpret the relationship between barrier injury and inflammatory progression in the context of altered mechanical forces.^10^, ^11^

Recent reviews have summarized Piezo1 and related Piezo channels from different perspectives, including their expression and function in the digestive system, gastrointestinal mechanosensation, disease‐associated roles, fibrosis, and interactions among intestinal forces, immunity and the microbiota.^2^, ^3^, ^12^, ^13^, ^14^ This review focuses on current evidence for Piezo1 in mechanotransduction, mucosal homeostasis, inflammatory responses and metabolism‐related phenotypes and signalling, while distinguishing between different cell types, experimental models, and species where possible. For findings that remain limited or inconsistent, we consider how cell state, mechanical stimuli, and disease phase may contribute to divergent outcomes. In metabolism‐related sections, we further distinguish functional metabolic evidence from signalling associations and hypotheses that remain to be tested. This approach provides a more cautious evaluation of the current evidence linking Piezo1 to metabolism‐related processes in digestive diseases.

### 1.1 Literature search strategy

PubMed/MEDLINE, Embase, Web of Science and Scopus were searched from database inception to 15 July 2026 using combinations of terms related to Piezo1, mechanotransduction, digestive diseases, inflammation, fibrosis and metabolism. English‐language primary studies with direct relevance to Piezo1 function in the digestive system were prioritized. Where findings were inconsistent, differences in cell type, experimental model, mechanical context, and disease phase were considered when interpreting the evidence.

## 2 FUNDAMENTAL MOLECULAR MECHANISMS OF PIEZO1 IN THE DIGESTIVE SYSTEM

### 2.1 Mechanosensing and Piezo1 Channel Activation

The digestive system is repeatedly exposed to dynamic mechanical stimuli generated by chyme propulsion and peristalsis, including luminal distension, tissue stretch, compression and fluid shear stress. Sensing and transducing these forces are essential for maintaining absorptive, secretory, barrier and motility functions.^3^, ^15^ Piezo1 is a major mechanosensitive cation channel participating in this process.^1^ Piezo1 rapidly converts external mechanical forces into changes in cation permeability and intracellular Ca^2+^ signalling.^1^, ^3^ Structurally, Piezo1 forms a trimeric, three‐bladed, propeller‐like architecture with a curved membrane dome that links membrane tension to opening of the central pore.^16^, ^17^ Beyond its ion‐channel function, Piezo1‐mediated conformational changes may also alter local membrane curvature and the behaviour of neighbouring membrane proteins, thereby influencing the spatial propagation of mechanical signals and subsequent cellular responses.^13^, ^18^

Piezo1 activation is not simply an instantaneous response to a single external force; rather, it reflects coordinated effects involving membrane tension, the membrane lipid environment, and structural constraints. Mechanical perturbation of the lipid bilayer can directly activate Piezo1, indicating a membrane‐dependent gating mechanism.^19^ Membrane lipid composition and lipid microdomains can further regulate Piezo1 conformational stability, mechanosensitivity, and inactivation kinetics, with cholesterol acting as an important modulatory factor.^4^ The cytoskeleton and extracellular matrix may also alter the activation threshold and functional output of Piezo1 by shaping membrane‐tension distribution, local structural support, and force transmission.^4^ Overall, Piezo1 gating and functional output are regulated across multiple levels by membrane lipid domains, cytoskeletal status, and the local cellular mechanical environment.^13^, ^17^

Piezo channels also contribute to gastrointestinal sensory mechanotransduction, but isoform attribution is essential. Piezo2 is a major epithelial and sensory‐neuronal mechanotransducer in several gut sensory pathways, whereas Piezo1 has distinct roles in enteric neurons and non‐neuronal digestive cell populations.^20^ In the digestive system, Piezo1 expressed in enteric neurons can sense mechanical stimuli and support colonic motility and immune homeostasis. Loss of Piezo1 function can lead to motility abnormalities, aggravated inflammation, and tissue injury.^21^ Piezo1 also contributes to intestinal smooth‐muscle function. In inducible smooth‐muscle‐cell‐specific Piezo1‐deficient mice, Piezo1 loss delayed gastrointestinal transit, impaired small‐bowel contractility, and altered intracellular Ca^2+^ activity within the intestinal muscularis.^22^ Another study demonstrated that Piezo1 activation can inhibit enteric neural crest cell migration, suggesting that Piezo1 may also participate in mechanosensitive responses during enteric nervous system development.^23^

Taken together, Piezo1 activation in the digestive system depends on the combined effects of mechanical stimuli, the membrane lipid environment, and structural constraints. Its principal significance lies in converting external mechanical changes into intracellular Ca^2+^ signals that initiate downstream biological and pathological responses.^3^, ^17^

### 2.2 Piezo1‐Mediated Ca^2+^ Influx and Downstream Signal Transduction

The direct biophysical consequence of Piezo1 opening is transmembrane cation flux, including Ca^2+^ entry.^1^ The resulting Ca^2+^ signal can engage calmodulin, kinases, proteases and transcriptional regulators.^24^ Its downstream effects depend on the amplitude, spatial distribution, and duration of Ca^2+^ signalling. Brief, spatially restricted Ca^2+^ signals may support adaptive mechanotransduction, whereas sustained Ca^2+^ loading has been associated in disease models with mitochondrial depolarization, reactive oxygen species accumulation, inflammasome signalling and cell death. These events should therefore be described as downstream, model‐dependent consequences of prolonged Piezo1 signalling rather than as intrinsic channel outputs. The following sections evaluate each pathway in relation to the relevant cell type, mechanical or inflammatory stimulus, disease model and Piezo1‐specific intervention.

#### 2.2.1 Epithelial Barrier Homeostasis, Mucus Protection and Mechanical Adaptation

In the intestinal epithelium, Piezo1 contributes to barrier regulation at multiple levels, including tight junction organization, mucus‐layer maintenance, epithelial junctional mechanics, and post‐injury repair. Intestinal barrier homeostasis requires coordinated regulation among the epithelium, mucosal immunity, and the vascular interface.^25^ Piezo1‐mediated Ca^2+^ signalling is closely involved in maintaining epithelial barrier homeostasis. Altered Piezo1 expression and activity can significantly affect epithelial permeability in Caco‐2 cells and mouse colonic epithelium. Piezo1‐mediated signalling also regulates the expression and distribution of claudin‐1, occludin, and zonula occludens‐1 via pathways involving Rho‐associated coiled‐coil‐containing protein kinase.^26^

In addition to tight junction‐dependent paracellular barrier regulation, Piezo1 participates in mucus‐layer maintenance through goblet‐cell mechanisms. Goblet cells and their secreted mucus layer form a physical barrier that contributes to epithelial renewal, immune segregation, and maintenance of the local microenvironment.^6^, ^27^ In mice, intestinal epithelial cell (IEC)‐specific Piezo1 deletion attenuated pressure‐induced colonic mucus secretion. In human LS174T goblet‐like cells, mechanical stimulation promotes mucin 2 (MUC2) expression through the Piezo1–extracellular signal‐regulated kinase–Ca^2+^ pathway, and Piezo1 knockdown or pharmacological inhibition attenuated the response, whereas Piezo1 activation produced the opposite effect.^28^ Additional studies in water‐avoidance stress mice have shown that Piezo1 activation in goblet cells can also upregulate MUC2 by suppressing suppressor of variegation 3‐9 homolog 1‐mediated histone H3 lysine 9 trimethylation. This pathway is therefore supported by combined mouse and cell evidence rather than established in humans.^29^

The mucus layer functions not only as a physical covering but also as an active interface in which mucin glycan composition, mucosa‐associated microbial colonization, and epithelial renewal are continuously coordinated. Changes in the mucus layer may therefore affect both microbial spatial distribution and host epithelial homeostasis.^30^, ^31^ In vivo studies have shown that goblet cell‐specific Piezo1 deletion decreases goblet cell numbers, reduces mucus‐layer thickness and remodels the mucosa‐associated microbiota. This remodelling is characterized by increased Firmicutes, decreased Verrucomicrobia and Actinobacteria, and greater abundance of *Helicobacter hepaticus, Lactobacillus johnsonii, Escherichia–Shigella*, and Oscillospiraceae‐related taxa within the inner mucus layer. These findings suggest that Piezo1 deficiency weakens the mucus barrier and promotes microbial enrichment on the mucosal side.^5^ In addition to mucus depletion, mice with goblet cell‐specific Piezo1 deletion also exhibit impaired epithelial homeostasis and delayed intestinal transit.^32^ These findings suggest that Piezo1 loss can alter both luminal content movement and epithelial renewal, and may further reshape the intestinal luminal microenvironment. Beyond tight‐junction and mucus‐layer regulation, intestinal epithelial homeostasis is also influenced by extracellular‐matrix mechanics, with matrix stiffness affecting epithelial adhesion, polarity and renewal balance.^33^, ^34^ Evidence from non‐digestive epithelial models further suggests that Piezo1 can support mechanical adaptation by balancing membrane and cortical tension, generating E‐cadherin‐coupled local Ca^2+^ signals, and promoting stretch‐induced cell division.^35^, ^36^, ^37^ These findings provide mechanistic plausibility for a similar role in the intestine, although direct evidence that Piezo1 mediates stiffness‐dependent intestinal epithelial responses remains limited.

**FIGURE 1:**
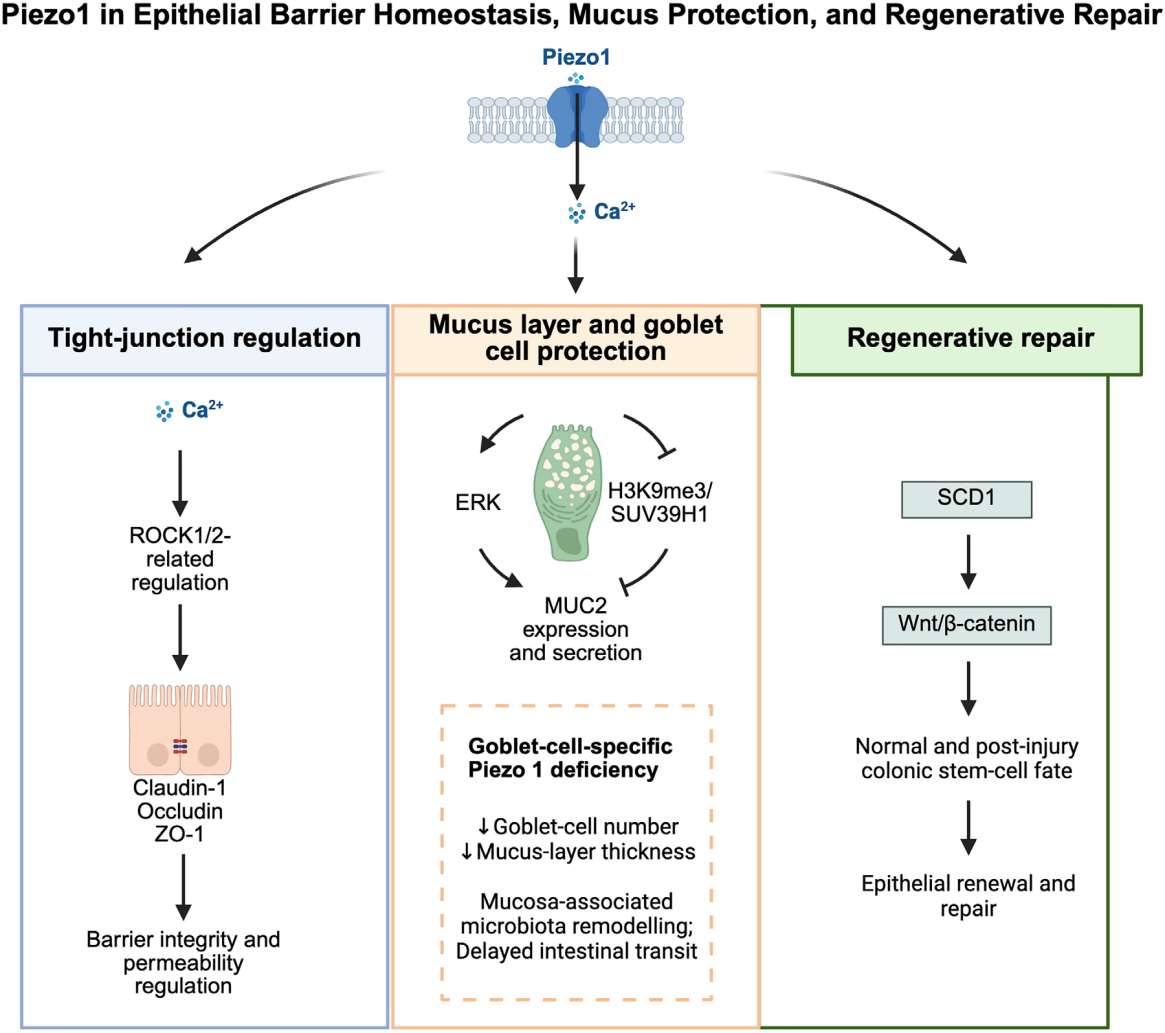
Piezo1‐mediated regulation of epithelial barrier homeostasis, mucus protection, and regenerative repair. This schematic summarizes the main mechanisms through which Piezo1 supports intestinal epithelial homeostasis. In intestinal epithelial cells, piezo1‐mediated Ca^2+^ signalling regulates claudin‐1, occludin, and zonula occludens‐1 (ZO‐1) through Rho‐associated coiled‐coil‐containing protein kinase (ROCK)‐related pathways, thereby supporting tight‐junction organization and permeability control. In goblet cells, piezo1 promotes mucin 2 (MUC2) expression and secretion through extracellular signal‐regulated kinase (ERK)‐ and histone H3 lysine 9 trimethylation (H3K9me3)/SUV39H1‐related mechanisms, whereas goblet‐cell‐specific Piezo1 deficiency reduces mucus protection and alters the mucosa‐associated microbiota. Piezo1 also regulates colonic stem‐cell fate through a stearoyl‐CoA desaturase 1 (SCD1)/Wnt/β‐catenin‐related programme, thereby supporting epithelial renewal and repair.

Recent evidence indicates that Piezo1 regulates normal and post‐injury colonic stem‐cell fate through stearoyl‐CoA desaturase 1 (SCD1)/Wnt/β‐catenin‐related programme. In vivo Piezo1 knockdown and activation altered colon stem‐cell state together with fatty‐acid‐related programmes in colonic crypts, while complementary mouse and human colitis‐organoid experiments further supported Piezo1‐dependent regulation of epithelial regeneration.^38^ Collectively, these findings suggest that Piezo1 supports intestinal barrier integrity through multiple coordinated processes, including junctional organization, mucus protection, epithelial mechanics and regenerative repair (Figure 1).

#### 2.2.2 Inflammatory Amplification and Mucosal Immune Activation

Here, inflammatory amplification refers to Piezo1‐dependent enhancement or persistence of an established inflammatory response, distinct from its initiation or basal mucosal immune surveillance. Under physiological mechanical conditions, Piezo1‐mediated Ca^2+^ signalling can support epithelial homeostasis, whereas excessive, persistent, or inflammation‐associated force can shift its output towards pro‐inflammatory signalling.^11^, ^39^

In dextran sulphate sodium (DSS)‐induced colitis in mice, Piezo1 inhibition limited group 3 innate lymphoid cell (ILC3) proliferation and decreased IL‐17A secretion.^40^ In ileal biopsies from patients with active Crohn's disease, epithelial Piezo1 expression was associated with disease activity and NOD‐, LRR‐ and pyrin domain‐containing protein 3 (NLRP3) signalling.^41^ Separate Yoda1, GsMTx4, and Piezo1‐knockdown experiments in human HT29 cells supported a Piezo1–Ca^2+^–mitochondrial stress–NLRP3 inflammatory pathway.^41^ Thus, Piezo1‐related inflammatory amplification involves both epithelial‐intrinsic signalling and immune‐cell‐dependent mechanisms.

Beyond epithelial cells, Piezo1 also contributes directly to myeloid cell‐driven amplification of mucosal inflammation. Piezo1 affects macrophage inflammatory mediator release, polarization states and mechanosensitive responses.^42^, ^43^ In chronic DSS colitis in mice, myeloid‐specific Piezo1 deletion reduced macrophage accumulation and M1‐like polarization, lowered levels of pro‐inflammatory mediators including TNF‐α, IL‐6 and IL‐1β, and alleviated disease severity, providing direct genetic evidence in that model.^44^ In mouse radiation‐induced colitis, Compound Kushen injection reduced macrophage Piezo1‐associated M1‐like polarization and nuclear factor‐κB (NF‐κB)/NLRP3 signalling. Macrophage‐specific Piezo1 ablation independently reduced M1‐like polarization and NF‐κB/NLRP3 activity and diminished the additional effect of Compound Kushen injection, supporting a macrophage Piezo1‐dependent mechanism.^45^

**FIGURE 2:**
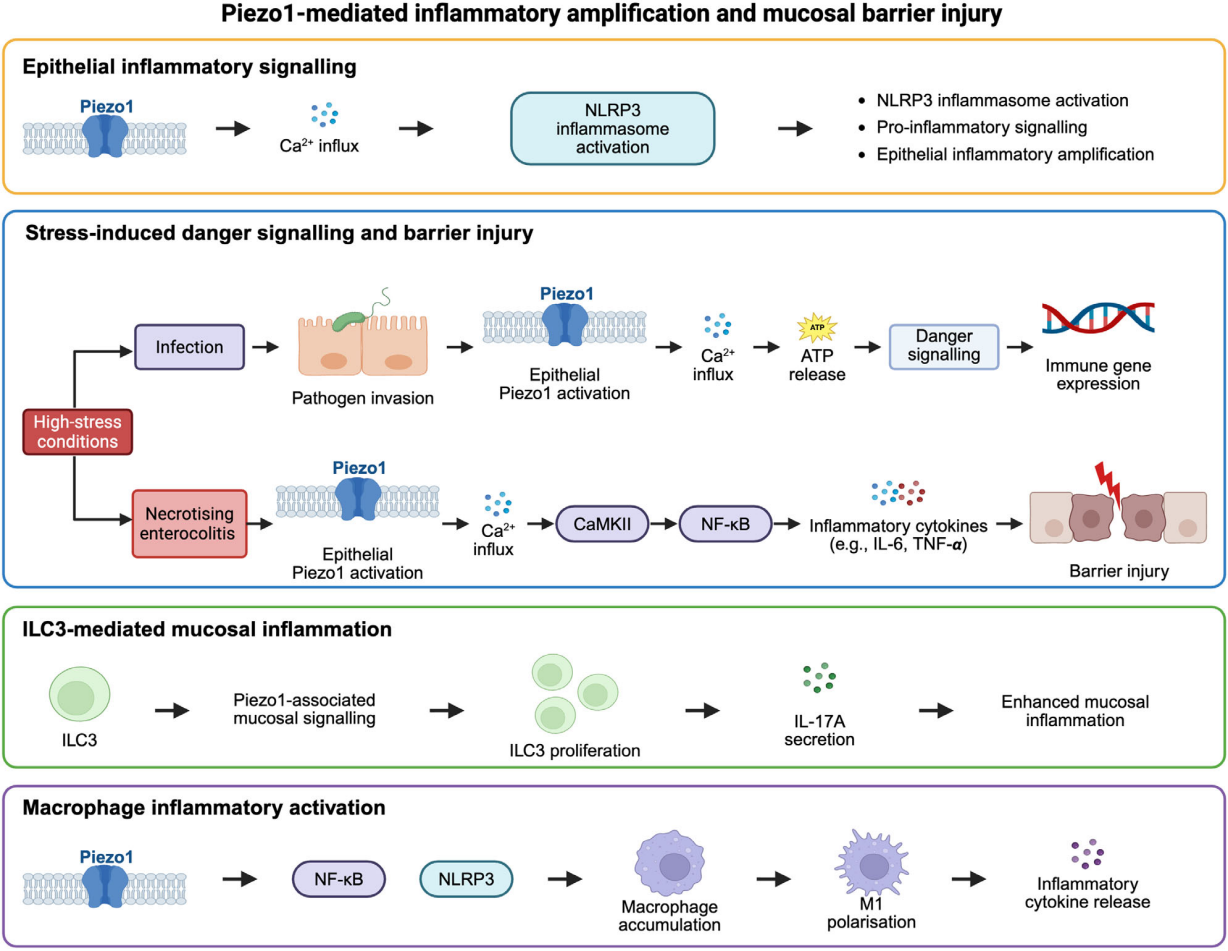
Piezo1‐mediated inflammatory amplification and mucosal barrier injury. This schematic summarizes the main pathways through which piezo1 contributes to intestinal inflammatory amplification and mucosal injury. In epithelial cells, piezo1‐mediated Ca^2+^ influx is associated with activation of the NOD‐like receptor family pyrin domain‐containing 3 (NLRP3) inflammasome and pro‐inflammatory signalling. Under high‐stress conditions such as infection, epithelial piezo1 activation promotes Ca^2+^ influx, adenosine triphosphate (ATP) release, danger signalling and immune gene expression. In necrotizing enterocolitis, Piezo1‐associated calcium/calmodulin‐dependent protein kinase II (CaMKII)/nuclear factor‐κB (NF‐κB) signalling promotes inflammatory cytokine release and barrier injury. Piezo1‐associated mucosal signalling also supports group 3 innate lymphoid cell (ILC3) proliferation and interleukin‐17A (IL‐17A) secretion, while in macrophages it contributes to inflammatory activation and cytokine release.

Under high‐stress conditions, Piezo1 can also amplify epithelial danger and inflammatory signalling. In IEC models of invasive bacterial infection, chemical and genetic Piezo1 inhibition reduced membrane‐ruffle‐induced Ca^2+^ entry and ATP release, demonstrating a Piezo1‐dependent response to invasion‐associated traction.^46^ In necrotizing enterocolitis, human neonatal tissue showed developmental and disease‐associated differences in epithelial Piezo1 expression. Causality was examined separately in neonatal IEC‐specific Piezo1‐knockout mice and human Caco‐2 cells, supporting a Piezo1‐mediated Ca^2+^ influx and calcium/calmodulin‐dependent protein kinase II (CaMKII)–NF‐κB inflammatory signalling.^47^ Collectively, these studies link Piezo1 to epithelial danger signalling, macrophage inflammatory responses and ILC3‐associated immune activation across distinct experimental models (Figure 2).

#### 2.2.3 Fibrotic Progression and Tumour Microenvironmental Change

Under sustained mechanical stimulation, inflammatory amplification and matrix stiffening, the biological effects of Piezo1–Ca^2+^ signalling may shift from acute responses towards extracellular‐matrix deposition, persistent cell‐state change, and tissue architectural distortion. Piezo1 is therefore considered here as a context‐dependent mechanotransduction component of fibrotic progression rather than a generic regulator of structural remodelling.^14^, ^48^

Pancreatic stellate cells are key extracellular‐matrix‐producing cells in chronic pancreatitis and pancreatic fibrosis and respond to both mechanical and inflammatory stimuli.^49^ Cell‐based studies show that Piezo1 contributes to stellate‐cell–matrix interactions.^50^ In pressure‐induced pancreatic fibrosis, conditional Piezo1 deletion in glial fibrillary acidic protein‐expressing stellate cells protected mice from fibrotic progression, whereas acinar‐cell‐specific Piezo1 deletion did not. Mechanical stress or Piezo1 activation promoted stellate‐cell activation through Piezo1‐dependent Ca^2+^ entry and subsequent transient receptor potential vanilloid 4 (TRPV4) signalling, increasing extracellular‐matrix‐related pro‐fibrotic responses.^50^, ^51^ Piezo1 also contributes to TRPC1/TRPV4‐dependent durotaxis along stiffness gradients, suggesting a role in the localization of pro‐fibrotic cells within stiffened tissue.^52^

The effect of Piezo1 on fibrosis varies with cellular state and disease phase. Macrophage‐specific Piezo1 deletion attenuated carbon tetrachloride‐ or bile duct ligation‐induced liver fibrosis by reducing inflammatory responses and cathepsin S secretion.^53^ By contrast, in a stiff matrix during early resolution, Piezo1 enhanced macrophage efferocytosis, fibrotic‐matrix clearance, and post‐engulfment anti‐inflammatory gene expression. Macrophage‐specific Piezo1 deletion impaired efferocytosis and the spontaneous resolution of early liver fibrosis.^54^ These findings are not mutually exclusive: the first study examines inflammatory macrophages during ongoing injury, whereas the second examines mechanically primed, pro‐resolving macrophage functions. Cell identity and activation state, mechanical context and injury versus resolution phase therefore influence the net outcome.^55^ In fibrotic intestinal tissues from patients with Crohn's disease, epithelial Piezo1 expression was increased. In DSS‐ and 2,4,6‐trinitrobenzenesulfonic acid‐induced chronic colitis models, IEC‐specific Piezo1 deletion attenuated intestinal fibrosis. In Crohn's disease‐related epithelial models, Piezo1 was linked to sustained hypoxia‐inducible factor‐1α (HIF‐1α) signalling, epithelial–mesenchymal transition, and fibrotic responses, supporting a separate epithelial route to chronic remodelling.^56^

In digestive tumours, Piezo1‐related pathways have been linked to invasion and stromal change, but the evidence remains model‐specific.^57^ In human gastric‐cancer tissues, Piezo1 abundance and Ca^2+^/HIF‐1α signalling were associated with omental metastatic phenotypes, while cell‐based intervention experiments supported effects on migration and calpain‐1/2.^58^ In *Helicobacter pylori*‐associated gastric‐cancer models, NF‐κB–Piezo1–Yes‐associated protein 1 (YAP1)–connective tissue growth factor signalling promoted cancer‐associated fibroblast infiltration, supporting tumour–stromal change.^59^ In hepatocellular‐carcinoma cell and xenograft models, Piezo1‐specific interventions supported transforming growth factor‐β‐dependent epithelial–mesenchymal transition.^60^ Other hepatocellular‐carcinoma studies reported mitogen‐activated protein kinase–YAP signalling or upregulation of C‐X‐C motif chemokine ligand 8 secretion through extracellular signal‐regulated kinase 1/2–protein kinase B signalling^61^, ^62^; these findings are not extrapolated beyond the tested tumour models.

**FIGURE 3:**
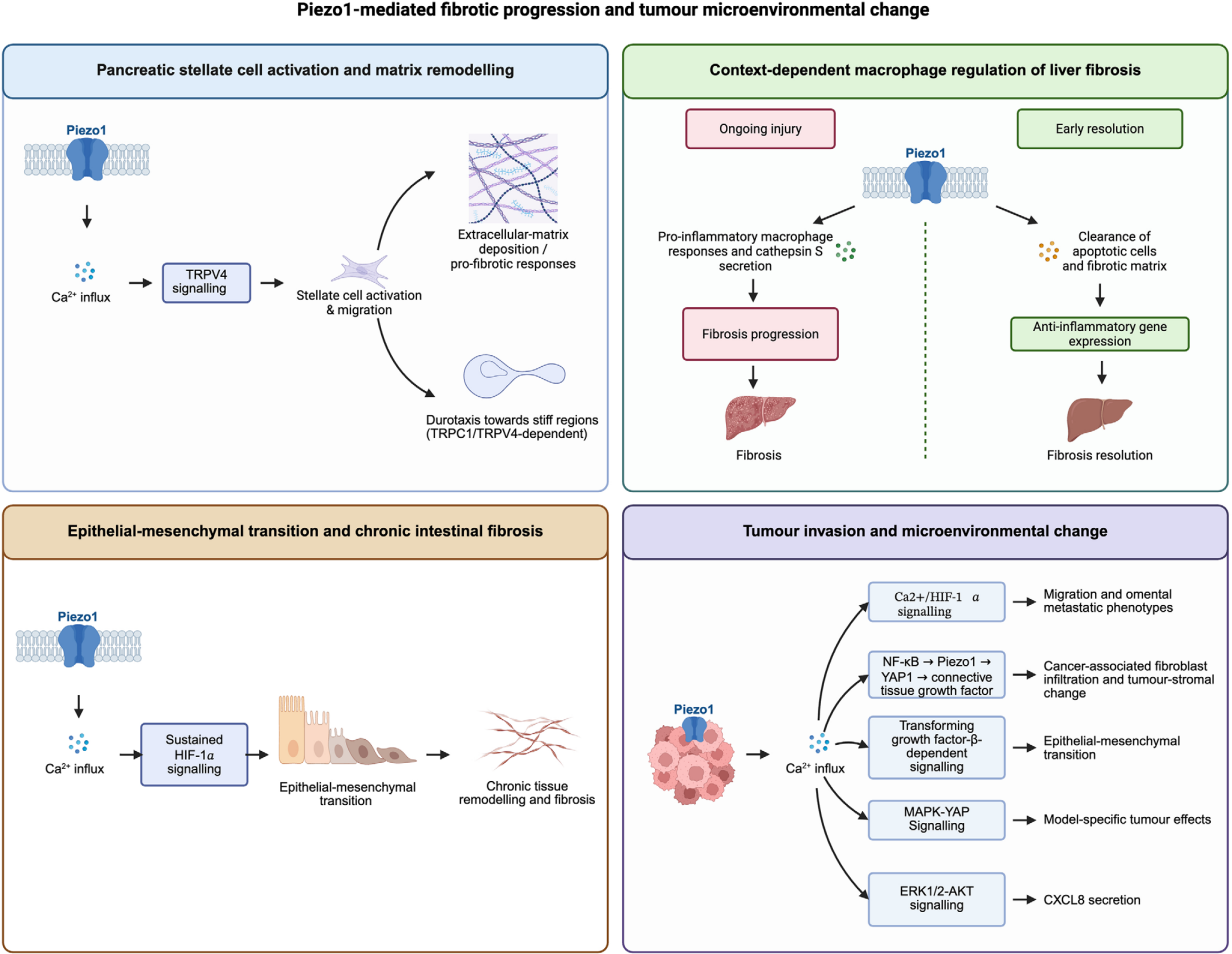
Piezo1‐mediated fibrotic progression and tumour microenvironmental change. This schematic summarizes the main pathways through which piezo1 contributes to fibrotic progression and tumour‐associated remodelling in digestive diseases. In pancreatic stellate cells, Piezo1‐mediated Ca^2+^ influx promotes transient receptor potential vanilloid 4 (TRPV4) signalling, stellate‐cell activation, migration, extracellular‐matrix deposition, and pro‐fibrotic responses, and also contributes to durotaxis through transient receptor potential canonical 1 (TRPC1)‐ and TRPV4‐dependent mechanisms. In liver fibrosis, piezo1 exerts context‐dependent effects in macrophages, promoting fibrosis progression during ongoing injury but supporting fibrosis resolution during early recovery. In chronic intestinal fibrosis, piezo1 is associated with sustained hypoxia‐inducible factor‐1α (HIF‐1α) signalling, epithelial–mesenchymal transition, and tissue remodelling. In tumour models, piezo1 has been linked to pathways involving HIF‐1α, nuclear factor‐κB (NF‐κB), Yes‐associated protein 1 (YAP1), transforming growth factor‐β, mitogen‐activated protein kinase (MAPK), extracellular signal‐regulated kinase 1/2 (ERK1/2), protein kinase B (AKT), and C‐X‐C motif chemokine ligand 8 (CXCL8), thereby contributing to invasion, stromal remodelling and tumour progression.

Overall, current experimental evidence supports distinct Piezo1‐dependent pathways across fibrotic and neoplastic settings rather than a single structural programme. In non‐neoplastic digestive disease, the principal outcomes include stellate‐cell activation, extracellular‐matrix deposition, and phase‐dependent macrophage functions. In tumour models, the reported outcomes include invasion‐associated cell states and cancer‐associated fibroblast recruitment (Figure 3).

#### 2.2.4 Mitochondrial Stress and Cellular Injury

Ferroptosis and mitochondrial dysfunction are important injury processes in digestive diseases.^63^, ^64^ Piezo1‐mediated Ca^2+^ influx has been associated with these outcomes across several disease models, although the strength of causal evidence varies across experimental settings.^65^, ^66^, ^67^ When Piezo1 activation is sustained or occurs under high‐stress conditions, Ca^2+^ signalling may shift from adaptive regulation towards overload‐associated injury, with mitochondrial depolarization, reactive oxygen species accumulation and loss of energy homeostasis reported in specific models.^65^, ^67^

In a mouse sepsis model, intestinal Piezo1 activation was accompanied by Ca^2+^ influx, loss of mitochondrial membrane potential, accumulation of reactive oxygen species, tight‐junction changes, and epithelial apoptosis; in caecal ligation and puncture‐induced sepsis, IEC‐specific Piezo1 deletion reduced intestinal permeability, tight‐junction disruption and epithelial apoptosis, providing direct genetic evidence for a causal role in barrier dysfunction.^65^ In mouse inflammatory bowel disease models, electroacupuncture and moxibustion altered Piezo1‐associated mitochondrial or ferroptosis‐related markers.^68^, ^69^ Because these studies used multi‐target interventions without Piezo1‐specific genetic rescue, they provide supportive rather than definitive evidence for Piezo1‐dependent injury.

In colonic tissue from patients with ulcerative colitis, epithelial Piezo1 abundance was associated with ferroptosis and tight‐junction disruption. Causality was examined separately in DSS‐treated IEC‐specific Piezo1‐knockout mice and lipopolysaccharide‐stimulated human Caco‐2 cells, where Piezo1 deletion or silencing reduced lipid peroxidation, altered ferroptosis‐associated proteins including GPX4, and improved mitochondrial and barrier measures, supporting involvement of AMP‐activated protein kinase (AMPK)/mechanistic target of rapamycin signalling.^66^ HT29 intervention experiments in the Crohn's disease study further supported Piezo1‐dependent Ca^2+^ loading and mitochondrial stress upstream of NLRP3 activation.^41^ One study reported higher Piezo1 expression in preterm neonatal specimens with lower gestational age and, in experimental necrotising enterocolitis models, supported a Piezo1–Ca^2+^/CaMKII–NF‐κB pathway.^47^ A separate study found increased epithelial Piezo1 expression in necrotising enterocolitis tissue. In experimental necrotising enterocolitis, IEC‐specific Piezo1 deletion protected epithelial barrier integrity, whereas pharmacological Piezo1 activation aggravated barrier disruption and disease severity. Complementary Caco‐2 experiments linked Piezo1‐dependent Ca^2+^ influx to endoplasmic‐reticulum stress, apoptosis, and junctional injury.^70^ Together, these studies support distinct Piezo1‐dependent injury mechanisms in necrotising enterocolitis rather than a single continuous signalling cascade.

**FIGURE 4:**
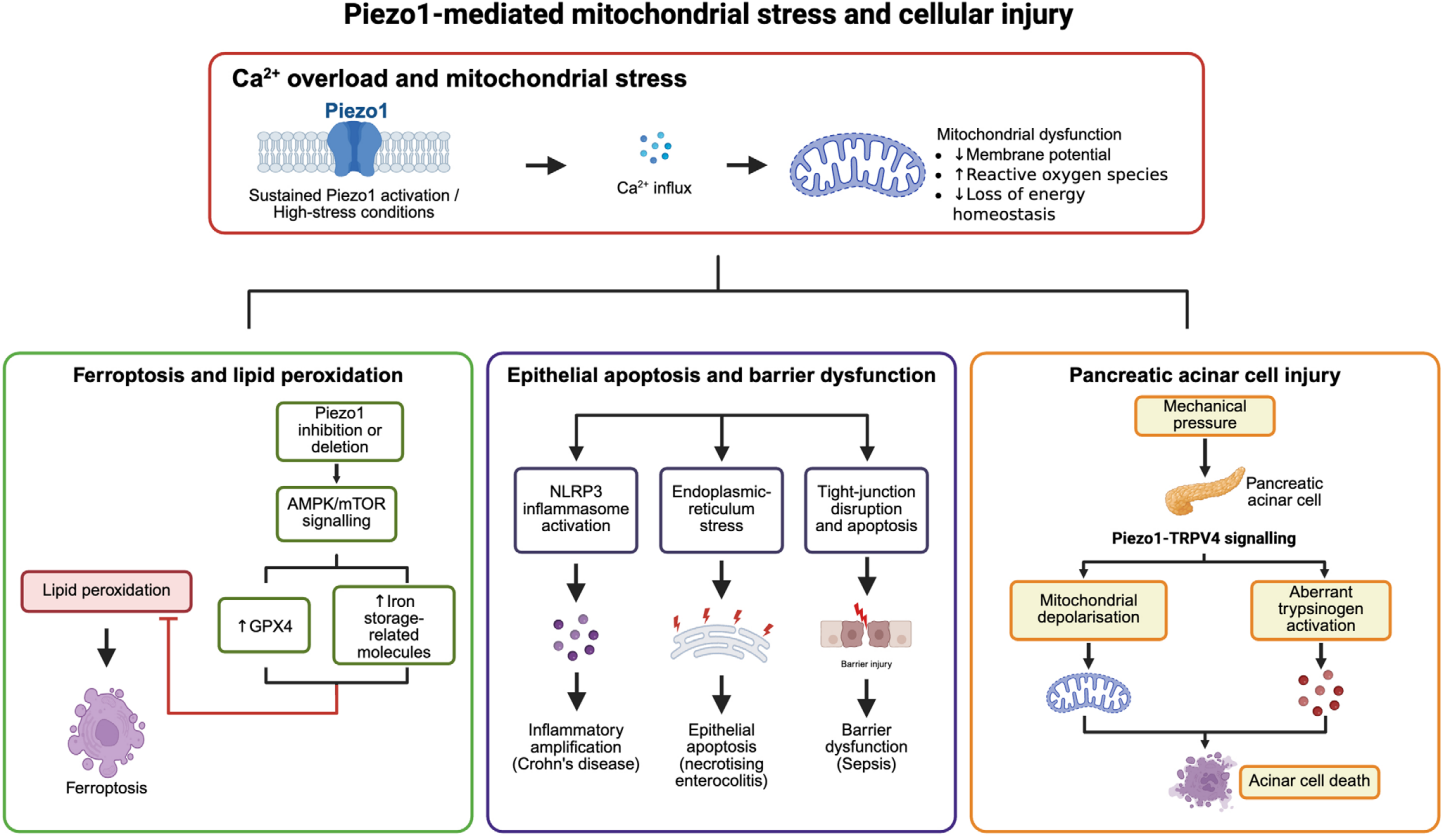
Piezo1‐mediated mitochondrial stress and cellular injury. This schematic summarizes the main pathways through which sustained piezo1 activation under high‐stress conditions contributes to mitochondrial dysfunction and downstream cellular injury in digestive diseases. Piezo1‐mediated Ca^2+^ influx is associated with reduced membrane potential, increased reactive oxygen species and loss of energy homeostasis. In intestinal epithelial injury models, piezo1 inhibition or deletion regulates AMPK/mTOR (AMP‐activated protein kinase/mechanistic target of rapamycin) signalling, restores glutathione peroxidase 4 (GPX4) and iron‐storage‐related molecules, and suppresses lipid peroxidation and ferroptosis. Piezo1‐associated epithelial injury is also linked to activation of the NOD‐like receptor family pyrin domain‐containing 3 (NLRP3) inflammasome, endoplasmic reticulum stress, and tight‐junction disruption with apoptosis, thereby contributing to inflammatory amplification and barrier dysfunction. In pancreatic acinar cells, mechanical pressure activates Piezo1/transient receptor potential vanilloid 4 (TRPV4) signalling, leading to mitochondrial depolarization, aberrant trypsinogen activation and acinar‐cell death.

This form of injury is not confined to the intestine. In a mouse pressure‐induced pancreatitis model and isolated pancreatic acinar cells, Piezo1 activation caused sustained Ca^2+^ elevation followed by mitochondrial depolarization, aberrant trypsinogen activation, and cell death. These Piezo1‐dependent injury responses were absent or markedly reduced in acinar cells from mice with acinar‐cell‐specific Piezo1 deletion, while TRPV4‐knockout mice were protected from Piezo1 agonist‐ and pressure‐induced pancreatitis, supporting a Piezo1–TRPV4 pathway.^67^ Across digestive models, Piezo1‐associated injury is therefore best viewed as context‐ and model‐dependent: sustained Ca^2+^ loading can precede mitochondrial dysfunction, lipid peroxidation, endoplasmic reticulum stress, or cell death, whereas transient signalling may remain adaptive (Figure 4). Table 1 summarizes the species and model for each evidence stream and grades causal attribution according to genetic intervention, rescue, pharmacology, or association.

**TABLE 1:** Piezo1‐related mechanisms and biological outcomes in digestive physiology and disease.

Disease or condition	Model/species	Evidence base	Piezo1‐related mechanism and outcome	Reference	Evidence tier
**Epithelial barrier homeostasis, mucus protection and mechanical adaptation**					
Intestinal barrier dysfunction	Caco‐2 cells; mouse colonic epithelium	ex vivo + in vitro	piezo1‐related Rho‐associated coiled‐coil‐containing protein kinase signalling; regulation of claudin‐1, occludin, and zonula occludens‐1 with altered epithelial permeability	^26^	B
Intestinal mucus homeostasis	IEC‐specific piezo1‐knockout mice; human LS174T goblet‐like cells	in vivo genetic + in vitro genetic/pharmacological	Pressure/mechanical piezo1–extracellular signal‐regulated kinase–Ca^2+^ signalling; colonic mucus secretion and MUC2 expression	^28^	A
Stress‐associated mucus regulation	Water‐avoidance stress mice; goblet‐cell models	in vivo + in vitro	Goblet‐cell piezo1 activation suppressed suppressor of variegation 3‐9 homolog 1‐mediated histone H3 lysine 9 trimethylation and increased MUC2 expression	^29^	A
Intestinal mucus‐barrier dysfunction	Goblet‐cell‐specific piezo1‐deficient mice	in vivo genetic	Piezo1 deficiency reduced goblet‐cell abundance and mucus thickness and altered the mucosa‐associated microbial community	^5^	A
Slow intestinal transit	Goblet‐cell‐specific piezo1‐knockout mice	in vivo genetic	Goblet‐cell piezo1 loss impaired epithelial homeostasis and delayed intestinal transit	^32^	A
Colonic epithelial injury and repair	Mouse colonic crypts; mouse and human colitis organoids	in vivo perturbation + organoid evidence	Piezo1 perturbation altered an SCD1/Wnt/β‐catenin‐related fatty‐acid programme, colonic stem‐cell fate, and epithelial regeneration	^38^	A
**Inflammatory amplification and mucosal immune activation**					
Experimental colitis	DSS‐induced colitis mice	in vivo pharmacological	Piezo1 inhibition limited ILC3 proliferation and decreased IL‐17A secretion	^40^	B
Chronic experimental colitis	Myeloid‐specific piezo1‐knockout DSS‐induced colitis mice	in vivo genetic	Myeloid piezo1 deletion reduced macrophage accumulation, M1‐like polarization, pro‐inflammatory mediators, and disease severity	^44^	A
Radiation‐induced colitis	Radiation‐induced colitis mice; macrophage‐specific piezo1 ablation	in vivo genetic + pharmacological	Macrophage piezo1‐dependent NF‐κB/NLRP3 signalling and M1‐like polarization; Compound Kushen injection effects were diminished after piezo1 ablation	^45^	A
Enteric bacterial invasion	IEC models of invasive bacterial infection	in vitro genetic + pharmacological	Chemical and genetic piezo1 inhibition reduced membrane‐ruffle‐induced Ca^2+^ entry and ATP release	^46^	C
Necrotizing enterocolitis	human neonatal intestinal tissue; neonatal IEC‐specific piezo1‐knockout mice; human Caco‐2 cells	human + in vivo genetic + in vitro	Disease‐associated epithelial piezo1 changes; piezo1–Ca^2+^–CaMKII–NF‐κB inflammatory signalling	^47^	A
Experimental colitis	Piezo1‐deficient mice; macrophage models under cyclical hydrostatic pressure	in vivo genetic + in vitro genetic/pharmacological	Macrophage piezo1–CaMKII–HIF‐1α signalling increased aerobic glycolysis; piezo1 deficiency reduced glycolytic activity, lactic acid abundance, and susceptibility to DSS colitis	^7^	A
**Fibrotic progression and tumour microenvironmental change**					
Pancreatic fibrosis	Pancreatic stellate‐cell models	in vitro	Piezo1 contributed to stellate‐cell–matrix interactions	^50^	C
Pressure‐induced pancreatic fibrosis	Conditional piezo1 deletion in glial fibrillary acidic protein‐expressing stellate cells; acinar‐cell‐specific piezo1 deletion	in vivo genetic	Mechanical stress/piezo1 activation promoted Ca^2+^‐dependent TRPV4 signalling and stellate‐cell activation; stellate‐cell piezo1 deletion protected against fibrotic progression	^51^	A
Pancreatic stromal stiffness	Pancreatic stellate‐cell stiffness‐gradient models	in vitro	Piezo1 contributed to TRPC1/TRPV4‐dependent durotaxis along stiffness gradients	^52^	C
Hepatic fibrosis	Macrophage‐specific piezo1‐knockout CCl_4_‐ or bile duct ligation‐induced fibrosis mice	in vivo genetic	Macrophage piezo1 deletion reduced inflammatory responses and cathepsin S secretion and attenuated liver fibrosis	^53^	A
Hepatic fibrosis resolution	Macrophage‐specific piezo1‐knockout fibrosis‐resolution mice; stiff‐matrix macrophage models	in vivo genetic + in vitro	Piezo1‐mediated stiffness sensing enhanced macrophage efferocytosis and matrix clearance; piezo1 deletion impaired spontaneous fibrosis resolution	^54^	A
Crohn's disease‐associated intestinal fibrosis	Human fibrotic Crohn's disease tissue; IEC‐specific piezo1‐knockout DSS/TNBS chronic colitis mice; epithelial models	human + in vivo genetic + in vitro	Increased epithelial piezo1 in fibrotic Crohn's disease; HIF‐1α‐associated epithelial–mesenchymal transition and fibrosis; IEC‐specific piezo1 deletion attenuated intestinal fibrosis	^56^	A
Gastric cancer with omental metastatic phenotypes	human gastric cancer tissue; gastric cancer cell models	human + in vitro	piezo1 abundance and Ca^2+^/HIF‐1α signalling were associated with omental metastatic phenotypes; cell interventions supported effects on migration and calpain‐1/2	^58^	B
*H. pylori*‐associated gastric cancer	*H. pylori*‐associated gastric cancer models	model‐specific experimental evidence	NF‐κB–piezo1–YAP1–connective tissue growth factor signalling promoted cancer‐associated fibroblast infiltration and tumour–stromal change	^59^	B
Hepatocellular carcinoma	Hepatocellular carcinoma cell and xenograft models	in vivo + in vitro	Piezo1‐specific interventions supported transforming growth factor‐β‐dependent epithelial–mesenchymal transition	^60^	B
Hepatocellular carcinoma	Hepatocellular carcinoma models	model‐specific experimental evidence	Piezo1 activation was linked to C‐X‐C motif chemokine ligand 8 secretion through extracellular signal‐regulated kinase 1/2–protein kinase B signalling	^61^	B
Hepatocellular carcinoma	Hepatocellular carcinoma models	Model‐specific experimental evidence	Piezo1‐related mitogen‐activated protein kinase–YAP signalling in tested tumour models	^62^	B
**Mitochondrial stress and cellular injury**					
Sepsis‐associated intestinal injury	IEC‐specific piezo1‐knockout caecal ligation and puncture mice	in vivo genetic	Intestinal piezo1 activation was associated with Ca^2+^ loading, mitochondrial depolarization, reactive oxygen species accumulation, junctional injury, and apoptosis; IEC‐specific deletion preserved barrier function	^65^	A
Experimental inflammatory bowel disease	Mouse inflammatory bowel disease models with electroacupuncture/moxibustion	in vivo multi‐target intervention	Multi‐target interventions altered piezo1‐associated mitochondrial or ferroptosis‐related markers without piezo1‐specific genetic rescue	^68^	D
Ulcerative colitis	Human ulcerative colitis tissue; DSS‐treated IEC‐specific piezo1‐knockout mice; lipopolysaccharide‐stimulated Caco‐2 cells	human + in vivo genetic + in vitro	Epithelial piezo1 was associated with ferroptosis and tight‐junction disruption; piezo1 deletion/silencing reduced lipid peroxidation and improved mitochondrial and barrier measures via AMPK/mechanistic target of rapamycin signalling	^66^	A
Crohn's disease	Human ileal biopsies; human HT29 cells	human + in vitro genetic/pharmacological	Epithelial piezo1 was associated with disease activity; piezo1 intervention supported a Ca^2+^–mitochondrial stress–NLRP3 inflammatory pathway	^41^	C
Necrotizing enterocolitis	Human necrotizing enterocolitis tissue; IEC‐specific piezo1‐knockout experimental necrotizing enterocolitis mice; Caco‐2 cells	human + in vivo genetic/pharmacological + in vitro	IEC‐specific piezo1 deletion protected barrier integrity, whereas piezo1 activation aggravated injury; Caco‐2 experiments linked piezo1‐dependent Ca^2+^ influx to endoplasmic‐reticulum stress, apoptosis, and junctional injury	^70^	A
Pressure‐induced acute pancreatitis	Acinar‐cell‐specific piezo1‐knockout mice; TRPV4‐knockout mice; isolated pancreatic acinar cells	in vivo genetic + ex vivo	Piezo1 activation caused sustained Ca^2+^ elevation, mitochondrial depolarization, trypsinogen activation, and cell death; piezo1‐ or TRPV4‐deficient models were protected	^67^	A

*Note*: human, human tissue or observational evidence; in vivo, animal studies; in vitro, cell or organoid studies; ex vivo, isolated‐tissue studies. Evidence tiers: A, direct piezo1‐specific causal evidence from in vivo functional perturbation with a direct outcome; B, piezo1‐specific in vivo or robust mechanistic evidence with an incomplete causal chain; C, in vitro mechanistic or human observational/correlative evidence without piezo1‐specific in vivo functional validation; D, indirect, multi‐target or hypothesis‐generating evidence lacking direct piezo1‐specific functional validation.

## 3 PIEZO1‐RELATED METABOLIC AND ENDOCRINE REGULATION IN DIGESTIVE DISEASES

**FIGURE 5:**
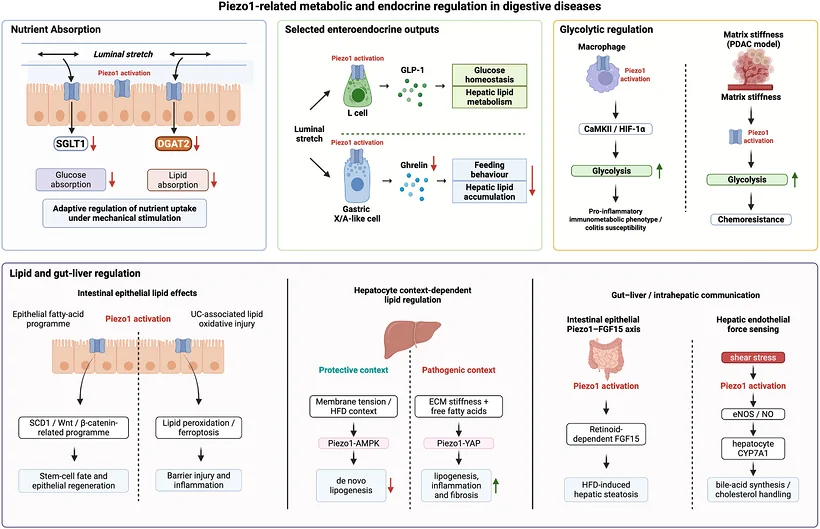
Piezo1‐related metabolic and endocrine regulation in digestive diseases. This schematic summarizes selected metabolic and endocrine effects of Piezo1 in the digestive system. In intestinal epithelial cells, Piezo1 activation reduces sodium‐glucose cotransporter 1 (SGLT1) and diacylglycerol O‐acyltransferase 2 (DGAT2), thereby limiting glucose and lipid absorption. In enteroendocrine cells, Piezo1 promotes glucagon‐like peptide‐1 (GLP‐1) output while suppressing ghrelin, linking mechanical stimulation to glucose homeostasis, feeding behaviour and hepatic lipid regulation. Piezo1 also promotes macrophage glycolysis through calcium/calmodulin‐dependent protein kinase II (CaMKII)/hypoxia‐inducible factor‐1α (HIF‐1α) signalling, whereas matrix stiffness enhances Piezo1‐associated glycolysis and chemoresistance in pancreatic ductal adenocarcinoma models. Lipid‐related effects are context‐dependent: intestinal epithelial Piezo1 influences fatty‐acid programmes, regeneration, lipid peroxidation and ferroptosis, while hepatocyte Piezo1 can exert protective or pathogenic metabolic effects depending on membrane tension or extracellular‐matrix stiffness. Gut–liver regulation further involves intestinal epithelial piezo1–fibroblast growth factor 15 (FGF15) signalling and hepatic endothelial piezo1‐dependent control of bile acid synthesis and cholesterol handling.

Piezo1‐mediated mechanosensing has been linked to metabolic and endocrine regulation in the digestive system. Current evidence is strongest when Piezo1‐specific perturbation is linked within the same model to a functional metabolic or endocrine endpoint, particularly in macrophage glycolysis, enterocyte nutrient handling, selected enteroendocrine outputs and hepatic lipid‐regulatory pathways. By contrast, evidence for Piezo1‐dependent lactate and lactylation pathways and for intestinal control of bile‐acid homeostasis remains limited or indirect. Accordingly, this section distinguishes functional metabolic and endocrine evidence from signalling associations and proposed cross‐system links rather than treating Piezo1 as a uniform mechanometabolic regulator (Figure 5).

### 3.1 Intestinal Epithelial Mechanosensing and Nutrient Absorption

Piezo1 contributes to intestinal nutrient handling and may adjust absorption to the mechanical state of the gastrointestinal lumen.^2^ Intestinal mechanosensing participates in postprandial signalling and coordinated nutrient and water absorption,^71^, ^72^ while tissue mechanics also influence epithelial morphology and homeostasis.^34^

In human duodenal samples from individuals with obesity, lower Piezo1–CaMKK2–AMPK signalling correlated with lipid accumulation.^73^ Causality was examined separately in high‐fat diet (HFD)‐fed IEC‐specific Piezo1‐knockout mice and human Caco‐2 cells. Intestinal epithelial Piezo1 deletion increased sugar and lipid absorption in mice, whereas stretch, Piezo1 overexpression, or Yoda1 reduced sodium‐glucose cotransporter 1, diacylglycerol O‐acyltransferase 2, and nutrient uptake in Caco‐2 cells.^73^ These findings provide direct experimental evidence for Piezo1‐dependent nutrient handling in mouse and cell models, whereas equivalent causal evidence in humans remains unavailable.

### 3.2 Gastrointestinal Endocrine Mechanosensing

Gastrointestinal mechanical stimuli can be translated into endocrine and neuroendocrine outputs through mechanosensitive secretory‐cell populations, although the responsible Piezo isoform varies by cell type and experimental model. In intestinal L cells, two mouse studies provide direct genetic evidence that Piezo1 couples luminal mechanical stimulation to glucagon‐like peptide‐1 (GLP‐1) output and systemic metabolism. L‐cell‐specific Piezo1 deletion reduced GLP‐1 production, worsened glucose tolerance, and disrupted hepatic lipid regulation, whereas intestinal bead implantation, tissue stretch, or Yoda1 produced opposite effects in control mice and mouse L‐cell models. These studies also supported CaMKKβ, CaMKIV, and mechanistic target of rapamycin complex 1 signalling in transgenic mice and mouse‐derived cells, but this pathway remains unvalidated in human L cells.^74^, ^75^

In human gastric samples, Piezo1 expression in X/A‐like cells was reduced in obesity but increased following sleeve gastrectomy.^76^ Causality was examined separately using X/A‐like‐cell‐specific Piezo1‐knockout mice, mechanical gastric distension induced by intra‐gastric bead implantation, pharmacological Piezo1 activation with Yoda1, and a ghrelin‐producing mouse cell line. These models supported a causal role for Piezo1 in suppressing ghrelin production and food intake through Ca^2+^‐dependent signalling.^76^ A separate ghrelin‐cell‐specific mouse study showed that Piezo1 deletion caused hyperghrelinaemia and hepatic steatosis, while pharmacological inhibition of the ghrelin receptor ameliorated hepatic steatosis, supporting a Piezo1–ghrelin mechanism linking gastric mechanosensing to hepatic lipid homeostasis.^77^

Anatomical evidence also identifies Piezo1 in murine antral gastrin‐producing G cells. Piezo1 was detected in most G cells and was enriched in the basolateral region near putative hormone‐release sites. This localization supports a potential role in G‐cell mechanosensing, but direct Piezo1‐dependent regulation of gastrin secretion has not been demonstrated.^78^ Enterochromaffin‐cell mechanotransduction requires isoform‐specific interpretation. Mechanical stimulation of enterochromaffin cells and related epithelial models supports rapid coupling between force sensing and neuroendocrine output.^79^ Pharmacological inhibition and Piezo‐family knockdown studies reported Piezo‐dependent effects on 5‐hydroxytryptamine (5‐HT) secretion, tryptophan hydroxylase 1 abundance, and intestinal motility, but did not establish Piezo1 as the principal enterochromaffin mechanosensor.^80^, ^81^ Under inflammatory conditions, Toll‐like receptor 4–Piezo1 signalling has been associated with altered 5‐HT responses and motility,^82^ but this should not be extrapolated to all enterochromaffin mechanosensation.

A previous study proposed that intestinal Piezo1 senses microbiota‐derived single‐stranded RNA and thereby regulates systemic 5‐HT synthesis.^83^ An independent re‐evaluation did not reproduce direct Piezo1 activation by the reported ribonucleic acid and found that cellular responses to faecal ribonucleic acid and extracts were Piezo1‐independent.^84^ More recent evidence from colorectal‐cancer models showed that bacterial extracellular‐vesicle‐derived single‐stranded ribonucleic acid suppressed tumour progression in a Piezo1‐dependent manner. Gut‐specific Piezo1 deletion and Piezo1‐deficient colorectal‐cancer cells attenuated this response, which was accompanied by reduced zinc finger protein 281 expression, lower leucine‐rich repeat‐containing G‐protein‐coupled receptor 5 abundance, and suppression of Wnt/β‐catenin signalling.^85^ However, these findings do not resolve whether microbial ribonucleic acid directly gates Piezo1. Independently of this proposed mechanism, the microbiota and its metabolites can influence 5‐HT production, whereas 5‐HT released by enterochromaffin cells contributes to local neural, immune, and metabolic signalling.^86^

Current evidence supports an asymmetric, multi‐step circuit rather than a closed Piezo1 feedback loop. In goblet‐cell‐specific Piezo1‐deficient mice, loss of Piezo1 reduced goblet‐cell abundance and mucus thickness and altered the mucosa‐associated microbial community.^5^ These concurrent changes support an association between Piezo1‐dependent mucus regulation and the microbial niche but do not establish that mucus loss caused the microbial shift. Separate gnotobiotic‐mouse and enterochromaffin‐cell studies showed that microbiota‐derived short‐chain fatty acids and other bacterial metabolites increased tryptophan hydroxylase 1 expression and 5‐HT biosynthesis.^87^, ^88^ Evidence that microbial metabolites or immune mediators alter epithelial Piezo1 expression, gating, or force sensitivity therefore remains lacking. Establishing such a return pathway will require cell‐specific Piezo1 perturbation, defined microbial communities, metabolomic profiling, calibrated mechanical stimulation and rescue experiments targeting mucus or candidate metabolites.

### 3.3 Glycolytic Regulation and Emerging Lactate‐Associated Pathways

Mechanical cues can alter cellular energy metabolism in digestive inflammation and tumours, but the strength of Piezo1‐specific evidence varies across experimental models. In macrophages, Piezo1‐specific genetic and pharmacological interventions link mechanosensation to increased aerobic glycolysis through CaMKII/HIF‐1α signalling. Under cyclical hydrostatic pressure, Piezo1 deficiency reduced glycolytic activity and lactic acid abundance, whereas Yoda1 increased glycolytic flux; Piezo1 deficiency also reduced susceptibility to dextran sulphate sodium‐induced colitis.^7^

In tumour models, the evidence is more heterogeneous. In three‐dimensional systems that model tumour stromal stiffening, matrix stiffness can enhance glycolysis through Piezo1 and is associated with chemoresistance in pancreatic ductal adenocarcinoma, whereas Piezo1 inhibition suppresses glycolysis and attenuates drug resistance.^89^ The same study linked Piezo1‐associated glycolysis to stiffness‐induced chemoresistance, although the evidence for Piezo1‐dependent lactate production remains pharmacological and model‐specific. In gastric cancer, Piezo1 upregulation increased Ca^2+^ signalling and HIF‐1α expression and promoted omental metastatic phenotypes.^58^ In hepatocellular carcinoma, matrix stiffness stimulated Piezo1 activity and decreased HIF‐1α ubiquitination, thereby enhancing angiogenic signalling,^90^ whereas a separate study linked Piezo1 activation to the metabolic regulator MTHFD2 and pro‐inflammatory CXCL8 signalling.^61^

Beyond the direct Piezo1–glycolysis evidence, lactate has diverse effects in digestive inflammation and cancer. In colitis models, lactate modulates macrophage polarization and mucosal repair,^91^ whereas pancreatic adenocarcinoma cells use monocarboxylate transporter 1‐mediated lactate uptake to withstand nutrient and oxidative stress.^92^ In pancreatic ductal adenocarcinoma, a glycolysis–histone lactylation feedback mechanism has been linked to tumour progression.^93^ More recent studies further show that matrix stiffness can increase lactate dehydrogenase A‐dependent lactate production and FOXO3 lactylation,^94^ or enhance glycolysis, lactate accumulation, histone H3 lysine 18 lactylation, and RAD51‐dependent radioresistance.^95^ Together, these findings define a plausible but unresolved interface between mechanical sensing, lactate metabolism, and lactylation in digestive disease. A key future question is whether Piezo1 couples pathological mechanical cues to lactate‐dependent signalling or lactylation in a cell‐ and disease‐specific manner. This will require Piezo1‐specific genetic perturbation combined with direct lactate measurements, extracellular flux or isotope‐tracing approaches, quantitative lactylation profiling, and rescue experiments targeting lactate production, transport, or candidate lactylated proteins.

### 3.4 Lipid Regulation and Gut–Liver Signalling

Piezo1‐related lipid regulation operates at multiple biological levels. Membrane lipids regulate Piezo1 gating, while Piezo1 signalling influences selected lipid‐related cellular processes and systemic metabolic pathways.

The membrane environment is an important determinant of Piezo1 activity. Mechanical stimulation of an isolated lipid bilayer can directly activate Piezo1, demonstrating an intrinsic membrane‐dependent gating mechanism.^19^ The curved membrane footprint of Piezo1 also increases its sensitivity to bilayer tension.^96^ Specific membrane lipids further modulate channel activity. Phosphoinositides and cholesterol can influence Piezo1 conformational stability, mechanosensitivity, channel clustering, and downstream signalling.^97^ Disturbing membrane cholesterol organization substantially decreases the activity of Piezo1 channel clusters.^98^ Piezo1 mechanosensitivity therefore reflects not only intrinsic channel properties but also membrane lipid composition, lipid microdomains and local membrane architecture.^4^, ^99^ In Caco‐2 cells, dietary compound‐induced changes in membrane cholesterol and fluidity were accompanied by altered Piezo1 expression and responses to shear stress.^100^ This finding supports an association between membrane lipid state and Piezo1 activity but does not establish that these compounds act specifically through Piezo1.

Beyond channel gating, Piezo1 has been linked to several local lipid‐related cellular effects. In mouse colonic crypts and mouse and human colitis organoids, Piezo1 perturbation altered stem‐cell fate together with changes in a SCD1/Wnt/β‐catenin‐related fatty‐acid programme.^38^ In human ulcerative colitis tissue, epithelial Piezo1 was associated with lipid peroxidation and ferroptosis. Epithelial‐specific Piezo1‐knockout mice with DSS‐induced colitis and lipopolysaccharide‐stimulated Caco‐2 cells provided further experimental support for this relationship.^66^ These findings suggest that Piezo1 can influence fatty‐acid‐related signalling and lipid oxidative injury in specific cellular and disease contexts.

Piezo1‐related lipid regulation also extends to gut–liver and intrahepatic signalling. In IEC‐specific Piezo1‐knockout mice, reduced retinol/retinoic‐acid‐dependent fibroblast growth factor 15 (FGF15) production attenuated HFD‐induced hepatic steatosis. FGF19 supplementation reversed this protective effect.^101^ These findings support a Piezo1–retinoid–FGF15 pathway in gut–liver lipid regulation rather than a classical bile acid–farnesoid X receptor (FXR) mechanism. Studies of L‐cell‐specific and ghrelin‐cell‐specific Piezo1 further linked mechanosensory endocrine signalling to hepatic lipid regulation.^74^, ^77^ In metabolic dysfunction‐associated steatotic liver disease, human tissue studies linked liver stiffness and Piezo1‐related signalling to disease‐associated features. Diet‐induced mouse models further supported an in vivo association between disease progression, tissue stiffening, and Piezo1‐related changes, but did not establish Piezo1‐specific causality. In cultured hepatocytes, stiff matrices combined with free‐fatty‐acid loading increased Piezo1 and YAP signalling, whereas GsMTx4 attenuated these responses.^102^

A distinct Piezo1‐related mechanism also operates within the liver. Using endothelial‐specific Piezo1 deletion and genetically enhanced Piezo1 activity, a mouse study showed that hepatic endothelial Piezo1 couples portal‐flow sensing to hepatocyte cholesterol 7α‐hydroxylase regulation through endothelial nitric oxide synthase/nitric oxide signalling. This pathway influences bile‐acid synthesis and cholesterol handling.^103^ Overall, current evidence suggests that Piezo1 may influence hepatic lipid homeostasis through several cell‐specific routes, including intestinal epithelial, enteroendocrine, and hepatic endothelial signalling.

Although the reported intestinal Piezo1–FGF15 effect was independent of classical bile acid–FXR signalling, the canonical FXR–FGF15/19–cholesterol 7α‐hydroxylase axis provides a potential framework for future investigation.^104^, ^105^ More broadly, gut–liver communication may provide a physiological context for investigating whether these Piezo1‐dependent pathways interact under specific metabolic or disease conditions.^106^ The current evidence linking Piezo1 to nutrient absorption, gastrointestinal endocrine signalling, glycolytic and lactate‐associated adaptation, and lipid and gut–liver regulation is summarized in Table 2.

**TABLE 2:** Piezo1‐related metabolic and endocrine regulation in digestive homeostasis and disease.

Research domain	Disease or condition	Model/species	Piezo1‐related mechanism	Major biological outcome	Reference	Evidence tier
Intestinal nutrient absorption	Obesity‐associated nutrient dysregulation	Human duodenal samples from individuals with obesity; HFD‐fed IEC‐specific piezo1‐knockout mice; human Caco‐2 cells	Reduced piezo1–CaMKK2–AMPK signalling in human tissue; piezo1‐dependent regulation of sodium‐glucose cotransporter 1, diacylglycerol O‐acyltransferase 2, and nutrient uptake	IEC‐specific piezo1 deletion increased intestinal sugar and lipid absorption; equivalent causal evidence in humans remains unavailable	^73^	A
Gastrointestinal endocrine mechanosensing	Hepatic lipid regulation	L‐cell‐specific piezo1‐knockout mice; mouse L‐cell models	piezo1‐dependent luminal mechanosensing and GLP‐1 output	L‐cell‐specific piezo1 deletion reduced GLP‐1 production and disrupted hepatic lipid regulation	^74^	A
	Glucose homeostasis	L‐cell‐specific piezo1‐knockout mice; transgenic mice and mouse‐derived L‐cell models	piezo1‐dependent Ca^2+^ signalling involving CaMKKβ, CaMKIV, and mechanistic target of rapamycin complex 1	L‐cell‐specific piezo1 deletion reduced GLP‐1 production and worsened glucose tolerance	^75^	A
	Obesity	Human gastric samples; X/A‐like‐cell‐specific piezo1‐knockout mice; gastric distension/Yoda1 models; ghrelin‐producing mouse cell line	X/A‐like‐cell piezo1‐dependent gastric mechanosensing and Ca^2+^ signalling	piezo1 suppressed ghrelin production and food intake; piezo1 expression in X/A‐like cells was reduced in obesity and increased after sleeve gastrectomy	^76^	A
	Hepatic steatosis	Ghrelin‐cell‐specific piezo1‐knockout mice; ghrelin‐receptor pharmacological intervention	piezo1–ghrelin mechanism linking gastric mechanosensing to hepatic lipid homeostasis	piezo1 deletion caused hyperghrelinaemia and hepatic steatosis; ghrelin‐receptor inhibition ameliorated steatosis	^77^	A
Glycolytic regulation	DSS‐induced colitis	Piezo1‐deficient mice; macrophage models under cyclical hydrostatic pressure	Macrophage piezo1–CaMKII–HIF‐1α signalling and aerobic glycolysis	Piezo1 deficiency reduced glycolytic activity, lactic acid abundance, and susceptibility to DSS‐induced colitis	^7^	A
	Pancreatic ductal adenocarcinoma	Three‐dimensional pancreatic ductal adenocarcinoma stromal‐stiffness models	Matrix stiffness‐associated piezo1 activation and enhanced glycolysis	Piezo1‐associated glycolysis was linked to stiffness‐induced chemoresistance; piezo1 inhibition reduced glycolysis and drug resistance	^89^	C
Lipid and gut–liver regulation	HFD‐induced hepatic steatosis	IEC‐specific piezo1‐knockout mice	piezo1‐dependent retinol/retinoic‐acid–FGF15 signalling	Reduced FGF15 production attenuated HFD‐induced hepatic steatosis; FGF19 supplementation reversed the protective effect	^101^	A
	Metabolic dysfunction‐associated steatotic liver disease	Human liver tissue; diet‐induced mouse models; cultured hepatocytes	Extracellular‐matrix stiffness‐associated piezo1–YAP signalling; GsMTx4‐sensitive responses in cultured hepatocytes	Disease progression and tissue stiffening were associated with piezo1‐related changes in vivo; cultured hepatocytes supported lipogenic, inflammatory, and fibrotic responses	^102^	C
	Metabolic dysfunction‐associated steatotic liver disease	HFD‐induced models; hepatocyte‐specific piezo1‐knockout mice	Increased hepatocyte membrane tension engages piezo1–AMPK signalling and suppresses de novo lipogenesis	Hepatocyte‐specific piezo1 deletion worsened hepatic triglyceride accumulation	^107^	A
Bile‐acid regulation	Physiological bile‐acid and cholesterol homeostasis	Endothelial‐specific piezo1 loss‐ and gain‐of‐function mouse models	Portal‐flow sensing through hepatic endothelial piezo1 and endothelial nitric oxide synthase/nitric oxide signalling regulates hepatocyte cholesterol 7α‐hydroxylase	piezo1‐dependent hepatic endothelial signalling influences bile‐acid synthesis and cholesterol handling	^103^	A

*Note*: Evidence tiers: A, direct piezo1‐specific causal evidence from in vivo functional perturbation with a direct outcome; B, piezo1‐specific in vivo or robust mechanistic evidence with an incomplete causal chain; C, in vitro mechanistic or human observational/correlative evidence without piezo1‐specific in vivo functional validation; D, indirect, multi‐target or hypothesis‐generating evidence lacking direct piezo1‐specific functional validation.

## 4 CONTEXT‐DEPENDENT PIEZO1 RESPONSES ACROSS CELL STATES, MECHANICAL STIMULI AND DISEASE PHASES

The biological direction of Piezo1 responses is not determined by channel activation alone. Transient physiological inputs that support homeostasis, secretion, barrier repair, or the resolution of inflammation generally favour adaptation. Sustained pressure, matrix stiffening, or traction, particularly alongside inflammatory or metabolic stress, instead favours tissue injury. Table 3 summarizes representative context‐dependent outcomes for which the direction of the net Piezo1 effect is supported by the available evidence, organised by cell identity and state, mechanical stimulus and tissue or disease phase.

**TABLE 3:** Context‐dependent Piezo1 responses across cell states, mechanical stimuli, and disease phases.

Cell type	Context‐defining condition	Net effect of piezo1	Reference	Evidence tier
**Epithelial cell identity and state**				
Intestinal goblet cells	Basal homeostasis or physiological mechanical stimulation	Protective—maintains mucus production, epithelial homeostasis and intestinal transit	^5^, ^28^, ^32^	A
Intestinal goblet cells	Water‐avoidance stress with pharmacological piezo1 activation by Yoda1	Protective—increases MUC2 expression under stress‐associated mucus dysregulation	^29^	A
Ileal epithelial cells	Active Crohn's disease; epithelial piezo1 associated with disease activity and NLRP3 signalling	Pathogenic association—linked to disease activity and piezo1‐dependent Ca^2+^–mitochondrial stress–NLRP3 inflammatory signalling	^41^	C
Colonic epithelial cells	Active ulcerative colitis and DSS‐induced colitis; IEC‐specific piezo1 deletion	Pathogenic—promotes lipid peroxidation/ferroptosis and barrier injury; deletion or silencing improves mitochondrial and barrier measures	^66^	A
**Neural and inflammatory cell identity and state**				
Enteric neurons	Physiological mechanosensing and piezo1 loss‐of‐function	Protective—supports colonic motility and immune homeostasis; loss aggravates inflammation and tissue injury	^21^	A
Intestinal macrophages	Cyclical hydrostatic pressure or Yoda1; DSS‐induced colitis	Pathogenic—increases CaMKII/HIF‐1α‐dependent aerobic glycolysis and colitis susceptibility	^7^	A
Intestinal macrophages	Chronic DSS‐induced colitis; myeloid‐specific piezo1 deletion	Pathogenic—sustains macrophage accumulation, M1‐like polarization, pro‐inflammatory mediators, and disease severity	^44^	A
**Disease phase and mechanical context**				
Hepatic macrophages	CCl_4_‐ or bile duct ligation‐induced liver fibrosis during the progression phase	Pathogenic—promotes inflammatory responses, cathepsin S secretion, and fibrotic progression	^53^	A
Hepatic macrophages	Stiffened matrix during the early fibrosis‐resolution phase	Protective—enhances efferocytosis and fibrotic‐matrix clearance and supports spontaneous fibrosis resolution	^54^	A
Hepatocytes	Lipid accumulation‐associated membrane tension in HFD‐induced MASLD; hepatocyte‐specific piezo1 deletion	Protective—piezo1–AMPK signalling suppresses de novo lipogenesis; deletion worsens hepatic triglyceride accumulation	^107^	A
Hepatocytes	Extracellular‐matrix stiffening with free‐fatty‐acid loading in MASLD	Pathogenic association with functional support—stiffness–piezo1–YAP signalling is linked to lipogenic, inflammatory, and fibrotic responses	^102^	C
**Metabolic cell identity and state**				
Intestinal L cells	Luminal mechanical stimulation; L‐cell‐specific piezo1 deletion	Protective—maintains GLP‐1 output, glucose tolerance, and hepatic lipid regulation	^74^, ^75^	A
Gastric X/A‐like/ghrelin cells	Gastric mechanical distension; X/A‐like‐ or ghrelin‐cell‐specific piezo1 deletion	Protective—suppresses ghrelin production and food intake and protects against hepatic steatosis	^76^, ^77^	A
Intestinal epithelial cells	HFD‐induced hepatic steatosis; IEC‐specific piezo1 deletion	Pathogenic under HFD—piezo1–retinoid–FGF15 signalling promotes hepatic steatosis; deletion lowers FGF15 and attenuates steatosis	^101^	A

*Note*: Net effect refers only to the stated experimental context. Evidence tiers: A, direct piezo1‐specific causal evidence from in vivo functional perturbation with a direct outcome; B, piezo1‐specific in vivo or robust mechanistic evidence with an incomplete causal chain; C, in vitro mechanistic or human observational/correlative evidence without piezo1‐specific in vivo functional validation; D, indirect, multi‐target or hypothesis‐generating evidence lacking direct piezo1‐specific functional validation.

During ongoing liver injury caused by CCl_4_ or bile duct ligation, macrophage Piezo1 worsens fibrosis by driving inflammation and cathepsin S secretion.^53^ During early resolution, however, Piezo1‐mediated stiffness sensing enhances macrophage clearance of apoptotic cells and fibrotic matrix and supports fibrosis resolution.^54^ Physiological stimulation of epithelial Piezo1 maintains goblet‐cell mucus secretion^5^, ^28^, ^29^ and epithelial homeostasis. Excessive or sustained epithelial Piezo1 signalling in models of active ulcerative colitis instead promotes lipid peroxidation, ferroptosis and barrier injury.^66^ Together, these results show that cell state and disease phase shape the net effect of Piezo1.^55^

Studies in hepatocytes further show that distinct mechanical cues can produce opposing outcomes within the same cell type and disease context. In HFD‐induced models of metabolic dysfunction‐associated steatotic liver disease, lipid accumulation raises hepatocyte membrane tension and engages Piezo1–AMPK signalling, thereby suppressing de novo lipogenesis. Hepatocyte‐specific Piezo1 deletion worsens hepatic triglyceride accumulation.^107^ By contrast, extracellular‐matrix stiffening combined with free‐fatty‐acid loading was associated with increased Piezo1 and YAP signalling in vivo, while cultured hepatocytes provided functional support for a stiffness–Piezo1–YAP mechanism linked to lipogenic, inflammatory and fibrotic responses.^102^ Together, these findings indicate that plasma membrane tension and extracellular matrix stiffness engage distinct downstream pathways under different metabolic conditions, yielding protective or pathogenic effects.

Overall, Piezo1 tends to support adaptation when its mechanosensory role matches cellular function and disease phase. Persistent activation alongside inflammation, lipotoxicity, or abnormal matrix stiffening can instead shift its effects towards pathology.

## 5 THERAPEUTIC IMPLICATIONS AND TRANSLATIONAL CHALLENGES

The available chemical tools are useful experimentally but are not yet disease‐ready therapeutics. Yoda1 is a Piezo1 agonist, and Jedi1/2 activate Piezo1 through an extracellular peripheral‐blade pathway.^108^, ^109^ Dooku1 opposes Yoda1‐induced Piezo1 activation but is not a validated broad inhibitor of mechanically activated Piezo1.^110^ GsMTx4 inhibits several mechanosensitive channels and cannot establish Piezo1 specificity; OB‐1/OB‐2 act indirectly through stomatin‐like protein 3‐dependent mechanotransduction and are not selective Piezo1 antagonists.^111^, ^112^

Because the effects of Piezo1 depend on cell type, mechanical context and disease phase, its activation or inhibition cannot be regarded as a uniform therapeutic strategy. Moreover, Piezo1 has broad expression across vascular, epithelial and sensory tissues; systemic modulation may therefore cause mechanism‐based on‐target adverse effects, while the limited selectivity of current tools may produce additional off‐target effects. Clinical translation will require more selective modulators, cell‐ or organ‐targeted or local delivery, and disease‐phase‐matched dosing strategies. Patient stratification should integrate the cellular localization of Piezo1, tissue stiffness, downstream pathway activity and disease phase rather than relying solely on total Piezo1 expression. No clinical evidence currently supports the efficacy of these compounds in Piezo1‐defined digestive disease indications.

## 6 LIMITATIONS AND FUTURE DIRECTIONS

This work is a narrative review rather than a systematic review. Although we applied a structured literature search, the review was not preregistered, and formal risk‐of‐bias assessment and meta‐analysis were not undertaken. The available evidence is derived predominantly from mouse, cell‐line and organoid models, whereas human studies are mainly observational. Studies also differ substantially in their mechanical stimulation paradigms, matrix stiffness, disease phase and intervention methods. Direct evidence remains limited or absent for Piezo1‐dependent lactate production, lactylation and intestinal bile acid regulation, while microbiota‐derived RNA sensing remains contested. These limitations preclude conclusions regarding uniform causality or immediate clinical translation.

Future studies should combine cell‐specific and inducible Piezo1 perturbation with standardised mechanical stimulation, disease‐phase stratification, rescue experiments and human tissue validation. Metabolic claims require confirmation through metabolic flux analysis, isotope tracing and direct metabolite quantification. Therapeutic studies should evaluate target engagement, Piezo1 versus Piezo2 selectivity, tissue exposure and effects on protective mechanosensory functions.

## 7 CONCLUSION

Overall, the significance of Piezo1 in digestive diseases extends beyond mechanosensing because the channel links changes in the mechanical microenvironment to barrier dysfunction, immune activation, tissue injury and remodelling, and selected metabolic or endocrine responses. The effects of Piezo1 are neither uniformly protective nor pathogenic; instead, they depend on cell identity and state, the profile and duration of mechanical forces and tissue and disease phase. Transient, phase‐appropriate activation may support tissue homeostasis and repair, whereas sustained activation during inflammatory or metabolic stress may exacerbate injury. Current mechanometabolic evidence most strongly supports cell‐specific effects of Piezo1 on macrophage glycolysis, enterocyte nutrient handling, and endocrine–lipid signalling. By contrast, evidence directly linking Piezo1 to lactate production, lactylation, or intestinal bile acid homeostasis remains limited or indirect. Piezo1 therefore represents a context‐dependent mechanotransducer rather than a universally actionable therapeutic target. Future studies should combine cell‐specific approaches, disease‐stage stratification, and human tissue validation to clarify the underlying mechanisms and therapeutic tractability of Piezo1. Successful clinical translation will require selective, tissue‐targeted, and temporally controlled modulation.

## AUTHOR CONTRIBUTIONS

Yuan Xia conceived the review, performed the main literature search, and drafted the manuscript. Chang Liang and Zhuoqing Zhuang, edited the manuscript. Zhe Xiong, Junzhu Lu and Pingrun Chen reviewed and approved the manuscript for publication. Yan Zhang supervised the work, acquired funding, and critically reviewed the manuscript. Yuan Xia, Chang Liang, and Zhuoqing Zhuang contributed equally to this work.

## CONFLICT OF INTEREST STATEMENT

The authors declare no conflicts of interest.

## ETHICS STATEMENT

The authors have nothing to report.

## Data Availability

No new datasets were generated or analysed for this narrative review. The bibliographic records and source studies used in the synthesis are publicly accessible through the databases and publications cited in the manuscript.
